# Onboard cone‐beam CT‐based replan evaluation for head and neck proton therapy

**DOI:** 10.1002/acm2.13550

**Published:** 2022-02-07

**Authors:** Alexander Stanforth, Liyong Lin, Jonathan J. Beitler, James R. Janopaul‐Naylor, Chih‐Wei Chang, Robert H. Press, Sagar A. Patel, Jennifer Zhao, Bree Eaton, Eduard E. Schreibmann, James Jung, Duncan Bohannon, Tian Liu, Xiaofeng Yang, Mark W. McDonald, Jun Zhou

**Affiliations:** ^1^ Department of Radiation Oncology and Winship Cancer Institute Emory University Atlanta Georgia USA; ^2^ New York Proton Center New York New York USA; ^3^ Department of Pre‐Medicine Cornell University New York New York USA; ^4^ Medical Physics Program Georgia institute of Technology Atlanta Georgia USA

**Keywords:** adaptive proton therapy, CBCT, deformable registration, ROC

## Abstract

**Purpose:**

Quality assurance computed tomography (QACT) is the current clinical practice in proton therapy to evaluate the needs for replan. QACT could falsely indicate replan because of setup issues that would be solved on the treatment machine. Deforming the treatment planning CT (TPCT) to the pretreatment CBCT may eliminate this issue. We investigated the performance of replan evaluation based on deformed TPCT (TPCTdir) for proton head and neck (H&N) therapy.

**Methods and materials:**

Twenty‐eight H&N datasets along with pretreatment CBCT and QACT were used to validate the method. The changes in body volume were analyzed between the no‐replan and replan groups. The dose on the TPCTdir, the deformed QACT (QACTdir), and the QACT were calculated by applying the clinical plans to these image sets. Dosimetric parameters’ changes, including ΔD95, ΔDmean, and ΔD1 for the clinical target volumes (CTVs) were calculated. Receiver operating characteristic curves for replan evaluation based on ΔD95 on QACT and TPCTdir were calculated, using ΔD95 on QACTdir as the reference. A threshold for replan based on ΔD95 on TPCTdir is proposed. The specificities for the proposed method were calculated.

**Results:**

The changes in the body contour were 95.8 ± 83.8 cc versus 305.0 ± 235.0 cc (*p* < 0.01) for the no‐replan and replan groups, respectively. The ΔD95, ΔDmean, and ΔD1 are all comparable for all the evaluations. The differences between TPCTdir and QACTdir evaluations were 0.30% ± 0.86%, 0.00 ± 0.22 Gy, and −0.17 ± 0.61 Gy for CTV ΔD95, ΔDmean, and ΔD1, respectively. The corresponding differences between the QACT and QACTdir were 0.12% ± 1.1%, 0.02 ± 0.32 Gy, and −0.01 ± 0.71 Gy. CTV ΔD95 > 2.6% in TPCTdir was chosen as the threshold to trigger QACT/replan. The corresponding specificity was 94% and 98% for the clinical practice and the proposed method, respectively.

**Conclusions:**

The replan evaluation based on TPCTdir provides better specificity than that based on the QACT.

## 1 INTRODUCTION

Intensity‐modulated proton therapy (IMPT) can spare more critical normal tissue than intensity‐modulated photon therapy for head and neck (H&N) cancer and hence the use of proton therapy is increasing.^1^, ^2^, ^3^

The highly modulated proton plans are sensitive to changes in patient anatomy such as tumor growth or shrinkage, weight loss, and cavity filling^1^, ^2^, ^3^ making a robust quality assurance (QA) process essential to the accurate treatment of the patient. Anatomical changes are particularly important in the treatment of H&N cancer patient as anatomical changes due to weight loss and tumor shrinkage or growth dramatically affect contours.^4^, ^5^, ^6^ Approximately 30% of H&N patients need to be replanned at least once during their course of IMPT treatment at Emory Proton Therapy Center.

**FIGURE 1:**
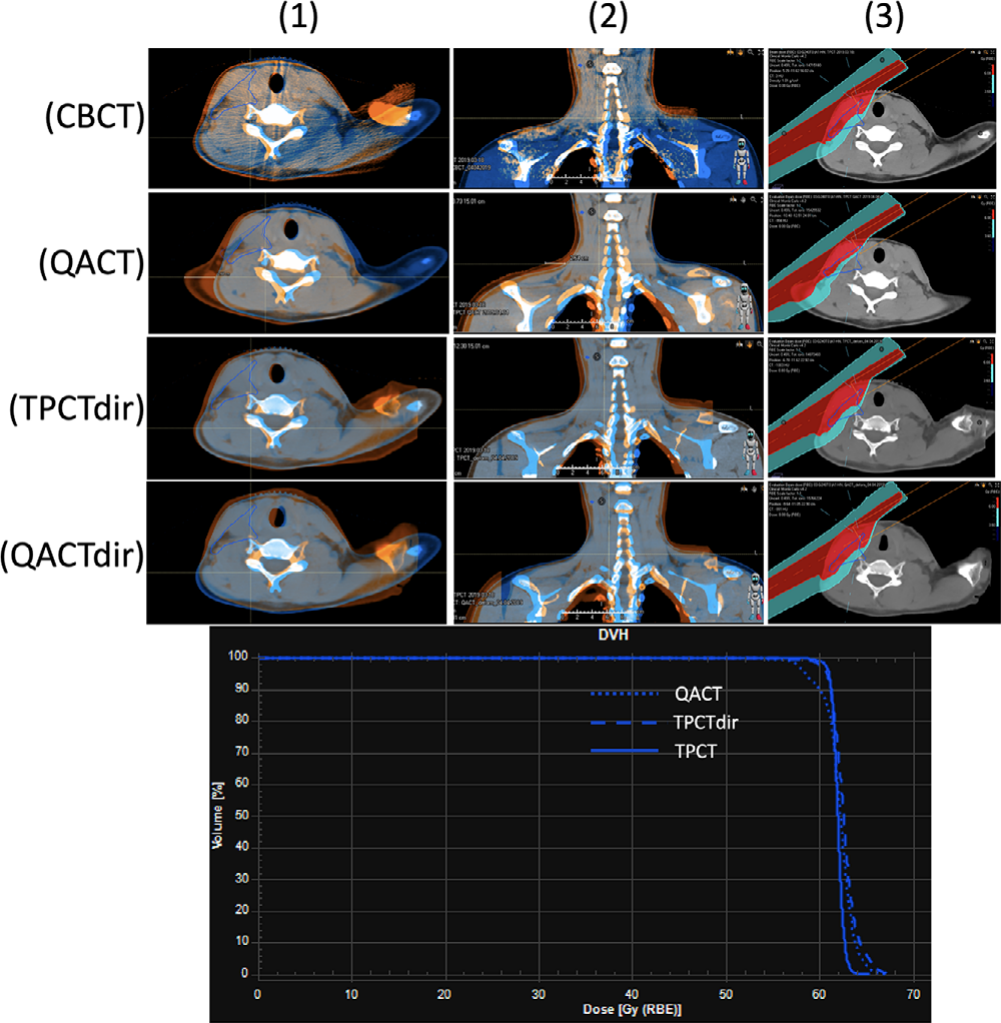
From top to the bottom are the image registration in axial (column 1), coronal (column 2), and their corresponding evaluation dose (column 3) from the posterior beam for the CBCT (row 1), QACT (row 2), TPCTdir (row 3), and QACTdir (row 4) with respect to the TPCT. The first row is from the initial nominal plan on the TPCT. The QACT shows a 2.64‐cm tissue difference at the neck region (second row) due to its inconsistent setup with the TPCT, while the CBCT, TPCTdir, and QACTdir match the TPCT better on the right (target) side. The dose volume histogram (DVH) plot (bottom) shows the incorrect node coverage reduction in the QACT (dotted blue line), correct evaluation in the TPCTdir (dashed line), and the initial coverage in the original plan (solid line). CBCT, cone‐beam CT; QACT, quality assurance CT; TPCT, treatment planning CT; TPCTdir, deformed TPCT to CBCT; QACTdir, deformed QACT to CBCT

The daily or weekly use of various CT systems such as CT‐on‐rails and in‐room mobile CT has been used for verifying the patient position, adaptive planning purposes, and calculating dose‐of‐the‐day.^7^ These setups have significant advantages but can greatly increase the time and complexity of the treatment process as well as adding significant cost to a center. QA CT (QACT) has also been routinely used in proton therapy practice but brings the risk of inaccurate setup. Patient positioning on the treatment machine is an iterative process, with multiple images necessary before the patient position is acceptable for treatment. QACT, which allows only one set of images, can falsely indicate need for replan because of setup issues that would be solved on the treatment machine. Although topogram (scout view) could possibly be used at the QACT to reproduce the patient position at the simulation, the lack of 2D image registration capability for most CT scanner software and typically overwhelmed CT scanner schedule prevent such technique to be effective. Figure 1 demonstrates the differences of shoulder position at treatment (shown in cone beam CT [CBCT]) and at QACT for an H&N patient. Deforming the treatment planning CT (TPCT) to the CBCT may eliminate this issue if the CBCT was obtained after accurate setup. Unfortunately, CBCT has poor hounsfield unit (HU) accuracy due to significant artifacts and is hence insufficient for dose calculation and dosimetric evaluation purposes. The recent development in deep learning‐based image quality improvement techniques has shown promising results for using the CBCT images directly in proton dose calculation.^8^, ^9^, ^10^ With these new techniques in imaging correction and faster dose calculation and optimization engine,^5^, ^11^ daily online adaptation could be feasible in the future.^2^ However, these techniques are neither accessible commercially nor currently available to most clinical settings.

A number of studies have been done on the accuracy of deformable image registration (DIR) between the planning CT and CBCT for H&N and lung cancers.^7^, ^10^, ^11^, ^12^, ^13^, ^14^, ^15^, ^16^ Veiga et al. examined the voxel‐wise dose accuracy and evaluated the change in water equivalent thickness (WET) for passive scattering proton treatment of lung cancer using CBCT deformable registration.^15^ A paper by Hou et al.^17^ and another one by Veiga et al.^12^ looked at these processes for H&N cancer using photons and found it can work well. Veiga et al. presented a workflow for adaptive radiation therapy using DIR in passive scattering proton therapy for lung treatments, in which the WET changes and range‐corrected planned dose were used for replan prediction.^18^ They also looked at three H&N patients being treated with IMPT to show that deformable registration could be used to compute the dose delivered.^19^ Though these studies provide useful knowledge regarding the accuracy of dose calculation based on the deformed TPCT, there is no report on the sensitivity and specificity of using such techniques for clinical decision‐making of replan in actual proton clinic for IMPT H&N treatments. Neither is there a report on using commercial software and workflow on such routine clinical practice. It has been suggested that TPCT images deformed to the CBCT could be used to indicate whether or not a replan or a QACT is necessary, reducing the number of CT scans a patient may undergo.^12^

In this study, we investigated the limitation of the QACT and the benefit of using a DIR of the TPCT to the CBCT (TPCTdir) in evaluating whether a replan was necessary for H&N patients treated with proton therapy. Commercially available clinical software packages were solely used for all analyses.

## 2 METHODS AND MATERIALS

### 2.1 Clinical data

Dose and imaging data for 28 H&N evaluations of 19 patients with a median age of 63 (range 14–84) who had undergone proton therapy treatments were analyzed. All patients were treated between December 2018 and August 2019 and had CBCT and QACT within 3 days. The dose prescription to the clinical target volumes (CTVs) ranged from 50.4 Gy relative biological effectiveness (RBE) to 70 Gy RBE (all doses referred in this paper are RBE dose). A detailed description of the patients can be found in Table 1. All bilateral H&N patients were planned with four beams (X shape configuration, two anterior and two posterior oblique beams) to five beams (with an additional anterior posterior [AP] beam). All cases were planned with robust optimization to CTVs in RayStation 8A (RaySearch Lab Inc., Stockholm, Sweden), using ± 3 mm setup in three orthogonal directions (and the nominal at 0 mm) and ± 3.5% range uncertainties (total 21 scenarios, 7 setup scenarios × 3 range scenarios [including the nominal at 0%]). Multifield optimization technique was used for all plans to spare the organs at risk (OARs). Sixteen of the 28 cases had undergone replan based on QACT evaluation (CTV ΔD95 > 3%) while the other 12 had not. The patient QACT and initial simulation scans were obtained using a Siemens SOMATOM Definition AS CT Scanner. All patients also had a CBCT taken with Varian's ProBeam on‐board imager before the treatment. Patients were immobilized with a 5‐point H&N masks (3 points on the head and 2 points on the shoulder) clipped onto the proton couch top (HP PRO Base Plates, Orfit, Belgium). H&N moldcare cushions were used to help setup reproducibility and to find the best comfortable treatment position. Hand holds were used and indexed to reproduce the shoulder positions at the treatment. CBCT scans were acquired using ProBeam's “Head” protocol, which consists of a 100 kVp, 154 mA beam taken in full‐fan mode, and half rotation. The field of view in this mode is 29 cm in diameter and 21.2 cm in superior/inferior direction. The CBCT system has a source‐to‐axis distance of 270 cm, helping reduce a significant amount of scatter in the CBCT. Literature indicates when comparing a CBCT to the QACT, it is best to use images taken within 3 days, so only those patients whose data met these criteria were used.^13^, ^20^

**TABLE 1:** Patient characteristics

				Rx to CTV1	Rx to CTV2	Rx to CTV3)	Rx to CTV4		
Patient identification (ID)	Age (y)	Gender	Classification of malignant tumors (TNM)	(Gy)	(Gy)	(Gy)	(Gy)	^#^Eval	^##^Replan
1	84	Male	T3N0M0	70	63	56	not available (N/A)	1	1
2	71	Female	T4N2Mx	70	59.85	53.9	N/A	2	2
3	66	Male	T4aN0M0	59.4	54	N/A	N/A	1	1
4	65	Male	T2N0M0	66	54	N/A	N/A	1	1
5	76	Male	T4bN3bM0	70	66.5	63	53.9	1	1
6	55	Male	T3N3M0	70	63	56	N/A	2	2
7	24	Female	T2N1M0	60	54	N/A	N/A	1	1
8	83	Male	T4bN0M0	60	45	N/A	N/A	1	1
9	57	Female	T2N2bM0	66	N/A	N/A	N/A	2	1
10	81	Male	T2N0M0	60	N/A	N/A	N/A	1	0
11	54	Female	T2bN1M0	60	N/A	N/A	N/A	4	0
12	35	Female	T4aN0M0	66	60	54	N/A	2	0
13	70	Female	T4N2M0	70	59.85	53.9	N/A	1	0
14	46	Male	T1N0M0	66	50	N/A	N/A	1	0
15	29	Male	T1N0M0	66	54	N/A	N/A	1	0
16	63	Male	T3N2M0	70	63	56	N/A	2	2
17	14	Male	T4N3M0	66	60	54	N/A	2	2
18	66	Male	T3N0M0	66	60	N/A	N/A	1	1
19	24	Female	T2N0M0	60	54	N/A	N/A	1	0

*Note*: Rx: prescription.

Abbreviation: CTV, clinical target volume.

^#^Eval: times of evaluation.

^##^Replan: times of replan.

### 2.2 Image registration

**FIGURE 2:**
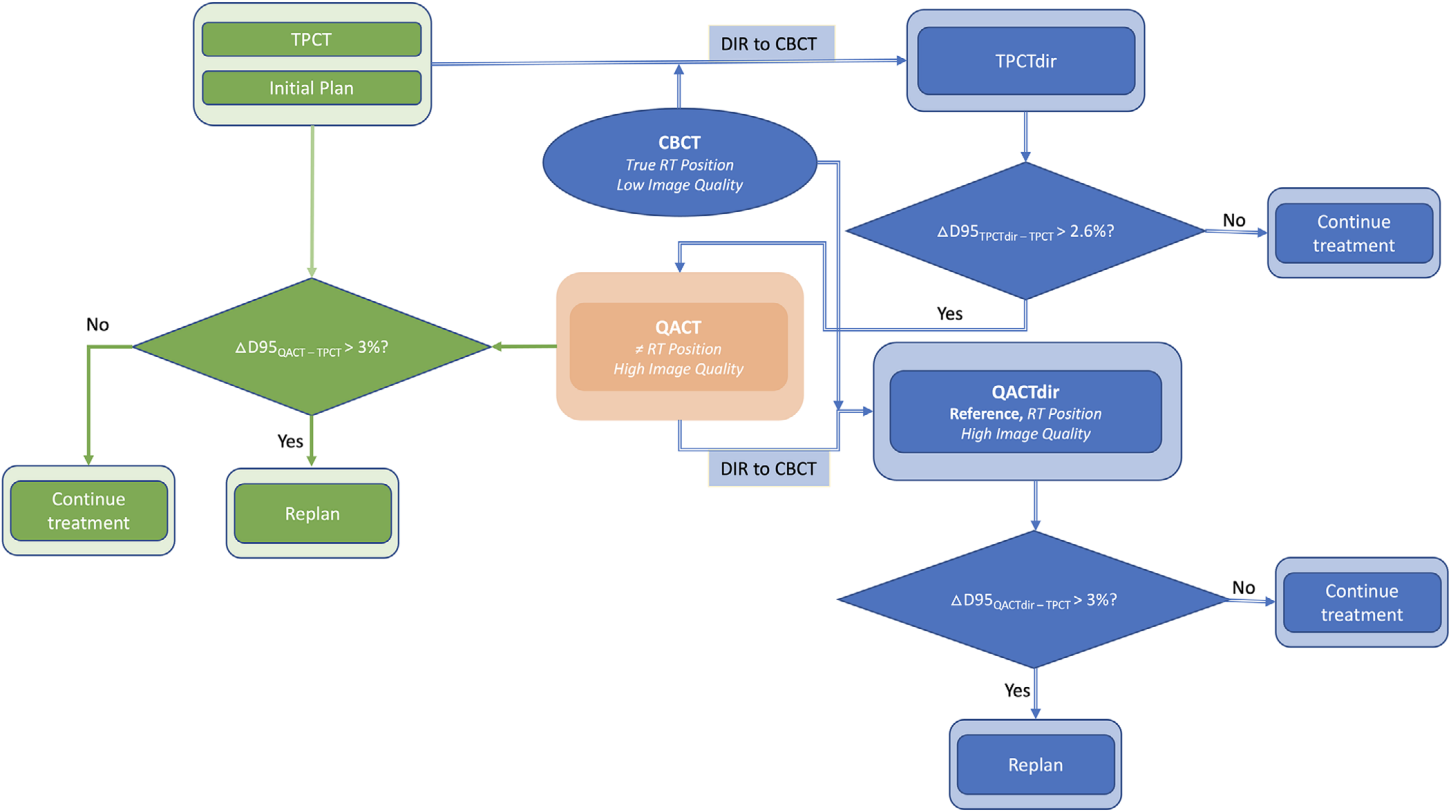
Clinical/conventional (green) and the proposed (blue) workflow for replan evaluation. Quality assurance CT (QACT, in orange) is involved in both workflows. CBCT, cone‐beam CT; DIR, deformable image registration; TPCT, treatment planning CT; TPCTdir, deformed TPCT to the CBCT

Figure 2 shows the workflow for both conventional and the proposed workflow for replan evaluation. Pretreatment onboard CBCT images were registered to the planning CT by experienced therapists before the treatment. These images represent the patient's actual anatomy and position for the treatment fraction. As the HU numbers in CBCT images are not accurate due to significant artifacts and scattering, the TPCT was deformed to the CBCT (TPCTdir) as an alternative for dosimetry evaluation. Varian's Velocity AI (version 4.1, Varian Medical Systems, Palo Alto California) was used to perform all DIR. The CBCT along with the online‐match rigid registration, which represents the patient treatment position relative to the TPCT, was sent to the Velocity. The CBCT correction algorithm built into Velocity AI was used to smooth the CBCT data in preparation for the DIR. A B‐spline algorithm built‐in to Velocity AI was used for the DIR from TPCTs to TPCTdirs (based on pretreatment CBCT). The same workflow was repeated for the QACT, with the exception of creating a new rigid registration as no online‐match registration is available. The deformed QACT (QACTdir) was used as a reference for dosimetric evaluation, as it represents both the treatment position and the most up‐to‐date patient's anatomy.

One of the most important factors in proton dose evaluation is to match the body surface shape (represented with the external body contour) between the evaluation image and the simulation (most often represented by the pretreatment CBCT). Body contour from the CBCT image is overlaid with the TPCTdir image to ensure their reasonably good agreement.

**FIGURE 3:**
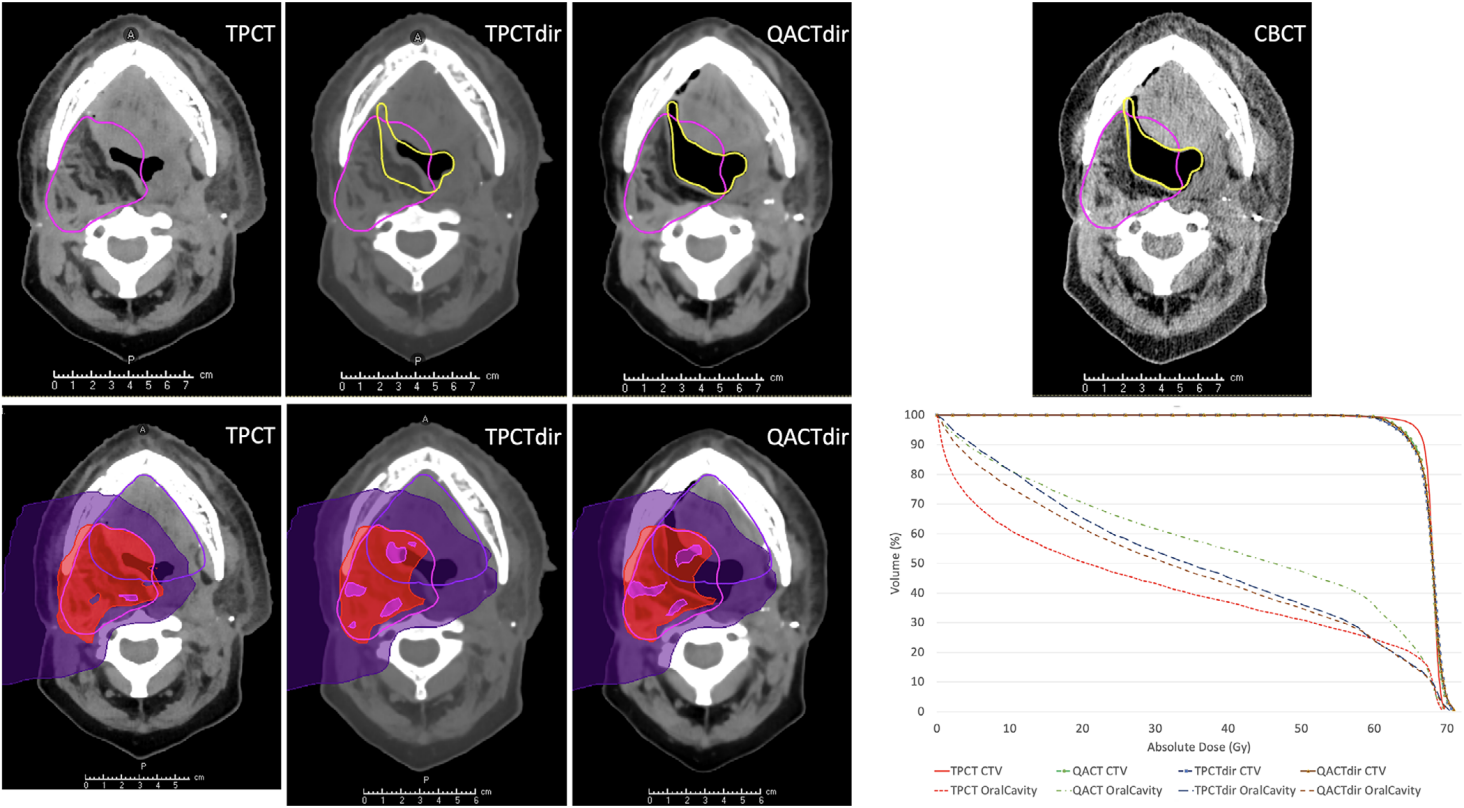
Shrinkage of graft and its effect on the oral cavity change. Treatment planning CT (TPCT), deformed TPCT to CBCT (TPCTdir), QACTdir, and the cone beam CT (CBCT) on top. The corresponding dose in the bottom (except for CBCT). The DVH for the clinical target volume (CTV) and the oral cavity are shown as well. Both QACTdir and the CBCT represent correct anatomy changes, while the TPCTdir failed to reflect the graft shrinkage. With density override to the air cavity contour derived from the CBCT, the dosimetric evaluation based on the TPCTdir matches that based on the QACTdir well. For demonstration purpose only, the CTV and oral cavity contours are rigidly copied (instead of deformed) from the TPCT to TPCTdir and QACTdir image sets, while the air cavity (yellow contour) is copied from CBCT to TPCTdir and QACTdir image sets

Some DIR required additional manual fine‐tuning. Smaller ROIs were used to encompass areas with disparities, and the deformation was continued to adequately model these differences. Additional corrections have been performed to account for cavity filling or emptying of sinuses as the DIR shows higher discrepancies in these regions. As discussed by Landry et al., a contour is used at areas that do not match and override with either air or muscle.^13^ Figure 3 demonstrates a TPCTdir failed to reflect the graft shrinkage that was on both the CBCT and the QACT. With density override to the air cavity contour derived from the CBCT, the dosimetric evaluation based on the TPCTdir matches that based on the QACTdir well. The contours of the targets and OARs were deformed and evaluated by a physician, and corrections were applied as needed. After the images were registered and corrected in Velocity AI, the resulting TPCTdir, QACTdir, and the corresponding DICOM RTstructures were sent to the treatment planning system (RayStation 8A).

### 2.3 Anatomy change evaluation

To quantify anatomical changes, the volume difference (cropped to the portion lying within the CBCT field of view (FOV)) of the body contours was calculated between the TPCT and the QACT, as well as between the TPCT and the TPCTdir. Receiver operating characteristic (ROC) analyses were conducted for the prediction of replan with both volume differences. In addition, the deformation from TPCT to CBCT was quantified by using the mean warp of the deformation field for the body contours using Velocity 4.1′s built‐in workflow for image deformation. An ANOVA analysis was conducted for the volume changes and the deformation warp between the no‐replan and replan group.

### 2.4 Dosimetric evaluation

The initial clinical treatment plan for the patient was used for dosimetric evaluation. The plan was applied to the TPCTdir, QACT, and QACTdir. The dose was calculated with a 3‐mm dose grid resolution and a clinical Monte Carlo dose engine to an uncertainty of 0.5%. A constant proton RBE of 1.1 was used for all cases. As many patient plans have several prescription dose levels and treated with simultaneous integrated boost technique, CTVs were divided into CTV_High (CTVs ≥ 59 Gy) and CTV_Low (CTVs < 59 Gy) categories and were evaluated separately, as high dose regions are typically in the more rigid head region while low dose regions are more in the flexible neck and shoulder regions. The dosimetric values including CTV D95, average dose, and D1 were evaluated, and the parotid mean doses were calculated on all the evaluation images. The differences of these dosimetric parameters in QACT and TPCTdir evaluation with respect to those in the corresponding reference QACTdir were evaluated. ANOVA analysis was used to compare the DVH parameters of the targets and OARs between different evaluations.

### 2.5 Dosimetric parameter ROC analyses

To evaluate the performance of the evaluation based on QACT and TPCTdir, an ROC analysis^21^, ^22^, ^23^ was conducted, using QACTdir as reference. The ROC curve is a plot of the true positive rate, or sensitivity (= TP/[TP + FN]) against the false positive rate (= 1 – specificity = FP/[FP + TN]) for each possible cutoff (here, the cutoffs are CTV ΔD95 at different levels). TP, false negative (FN), false positive (FP), and true negative (TN) are numbers of (1) true positives, (2) false negatives, (3) false positives, and (4) true negatives, respectively. In replan evaluation, they represent the cases that the evaluation (1) predicts correctly for replan, (2) predicts incorrectly for no‐replan, (3) predicts incorrectly for replan, and (4) predicts correctly for no‐replan. A good evaluation is associated with high sensitivity (miss few patients that do need replan) and specificity (few false alarms for patients who do not need replan, which waste staff resources). Furthermore, the performance of the evaluations is measured by calculating the region area under the ROC curve (AUC).

ΔD95 in both QACT and TPCTdir evaluations were calculated and used as test variables. A ΔD95 > 3% in QACTdir was deemed as a true replan and used as the state variable. The sensitivity and specificity of the evaluation using QACT evaluation were calculated. A threshold value for replan from ΔD95 evaluation using the TPCTdir is recommended. All the analyses were implemented in SPSS V25 (IBM Inc, Chicago, IL). A *p*‐value of <0.05 is considered statistically significant.

## 3 RESULTS

### 3.1 Anatomy changes evaluation

**FIGURE 4:**
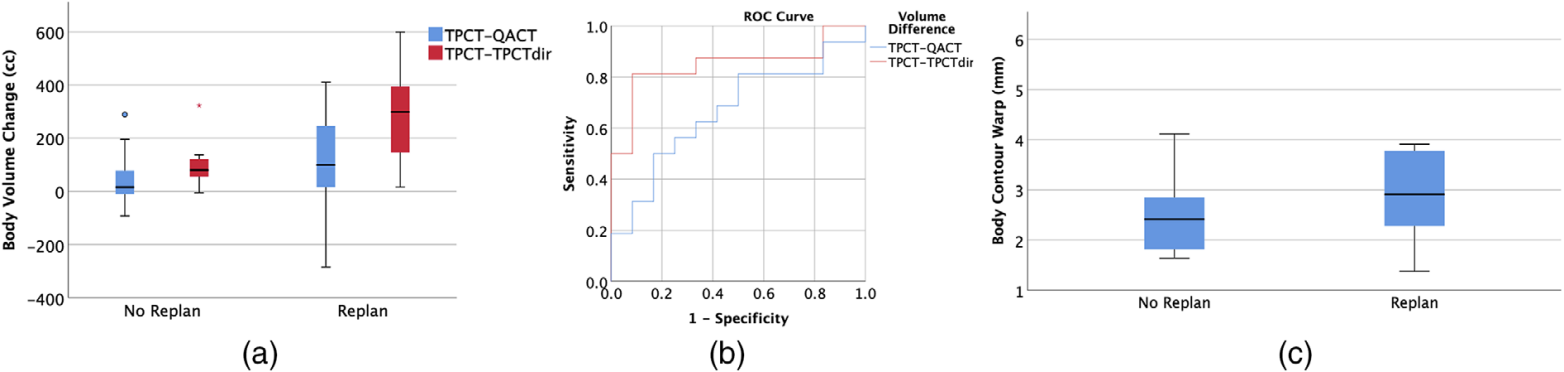
(a) Box plot of body contour volume difference from treatment planning CT (TPCT) to quality assurance CT (QACT) (blue), and TPCT to TPCTdir (red), for no‐replan and replan groups; (b) receiver operating characteristic (ROC) curves for using TPCT ‐ QACT and TPCT ‐ TPCTdir to predict replan; and (c) box plot of mean deformation warp between no‐replan and replan group

Overall, the mean differences in the body contour within CBCT FOV between the TPCT and QACT were 43.4 ± 109.5 cc versus 111.1 ± 172.6 cc (*p* = 0.25) for the no‐replan and replan patient groups, respectively. The corresponding difference between TPCT and TPCTdir was 95.8 ± 83.8 cc versus 305.0 ± 235.0 cc (*p* < 0.01). Figure 4b shows the ROC curves for using volume difference of TPCT‐QACT and TPCT‐TPCTdir to predict replan. The AUCs were 0.67 and 0.85, respectively. The mean warps for the body contour were 2.5 ± 0.8 mm versus 5.2 ± 9.6 mm (*p* = 0.34) for the no‐replan and replan groups, respectively. Their distributions are shown in Figure 4c.

### 3.2 Dose distribution

**FIGURE 5:**
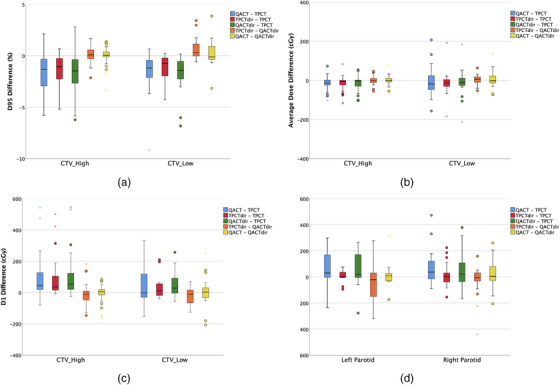
Boxplots of the differences in D95 (a), average dose (b), and D1 (c) for both clinical target volume (CTV) groups and average dose (d) for both parotids between the quality assurance CT (QACT) and treatment planning CT (TPCT), TPCTdir and TPCT, QACTdir and TPCT, TPCTdir and QACTdir, QACT and QACTdir. The circles and the asterisks are outliers (at least 1.5 box lengths from the median) and extremes (at least three box lengths from the median), respectively

There were 43 CTV_Highs, 20 CTV_Lows, and 35 parotids evaluated. The mean differences between different evaluations (QACT vs TPCT, TPCTdir vs TPCT, QACTdir vs TPCT, TPCTdir vs QACTdir, and QACT vs QACTdir) in D95, average dose, and D1 for CTV_High, CTV_Low, and average dose for the parotids are summarized in Table 2. The corresponding statistical distributions are shown with a box plot in Figure 5. As QACTdir was deemed the reference for the evaluation, the reference dosimetric degradation, which is represented by QACTdir – TPCT shows that CTV_Highs have a trend toward less D95 change and less variability than those from CTV_Lows, −1.68% ± 1.77% versus −2.86% ± 5.36% (*p* = 0.2, not statistically significant). The mean differences of both CTVs between the TPCTdir and the reference QACTdir were 0.30% ± 0.86%, 0.00 ± 0.22 Gy, and −0.17 ± 0.61 Gy, for D95, Dmean, and D1, respectively (not shown in Table 2). The corresponding differences between QACT and QACTdir were 0.12% ± 1.1%, 0.02 ± 0.32 Gy, and −0.01 ± 0.71 Gy. The mean differences for the parotids with respect to QACTdir were −0.33 ± 1.28 Gy and 0.22 ± 1.08 Gy for TPCTdir and QACT, respectively. The corresponding parotid mean difference between TPCTdir and QACTdir was −0.33 ± 1.28 Gy (*p* = 0.3, not statistically significant).

**TABLE 2:** Differences of dosimetric parameters in different evaluations

Dose difference inevaluations	D95 (%)	Mean Dose (Gy RBE)	D1(Gy RBE)	Mean dose(Gy RBE)
	CTV_High (*n* = 43)/CTV_Low (*n* = 20)			Parotid (*n* = 35)
QACT ‐ TPCT	−1.65 ± 1.58 / −2.56 ± 4.68	−0.15 ± 0.34/−0.05 ± 0.81	0.87 ± 1.20/0.89 ± 2.26	0.66 ± 1.36
TPCTdir ‐ TPCT	−1.55 ± 1.51 / −2.20 ± 4.96	−0.15 ± 0.33/−0.12 ± 0.65	0.78 ± 1.08/0.60 ± 1.64	0.10 ± 0.77
QACTdir ‐ TPCT	−1.68 ± 1.77 / −2.86 ± 5.36	−0.15 ± 0.35/−0.13 ± 0.73	0.91 ± 1.25/0.85 ± 1.80	0.44 ± 1.35
TPCTdir ‐ QACTdir	0.13 ± 0.69 / 0.66 ± 1.07	−0.01 ± 0.20/0.01 ± 0.27	−0.13 ± 0.63/‐0.24 ± 0.57	‐0.33 ± 1.28
QACT ‐ QACTdir	0.03 ± 0.82/0.30 ± 1.56	−0.01 ± 0.22/0.08 ± 0.47	−0.04 ± 0.55/0.04 ± 1.00	0.22 ± 1.08

Abbreviations: CTV, clinical target volume; QACT, quality assurance CT; TPCT, treatment planning CT.

### 3.3 ROC analysis

**FIGURE 6:**
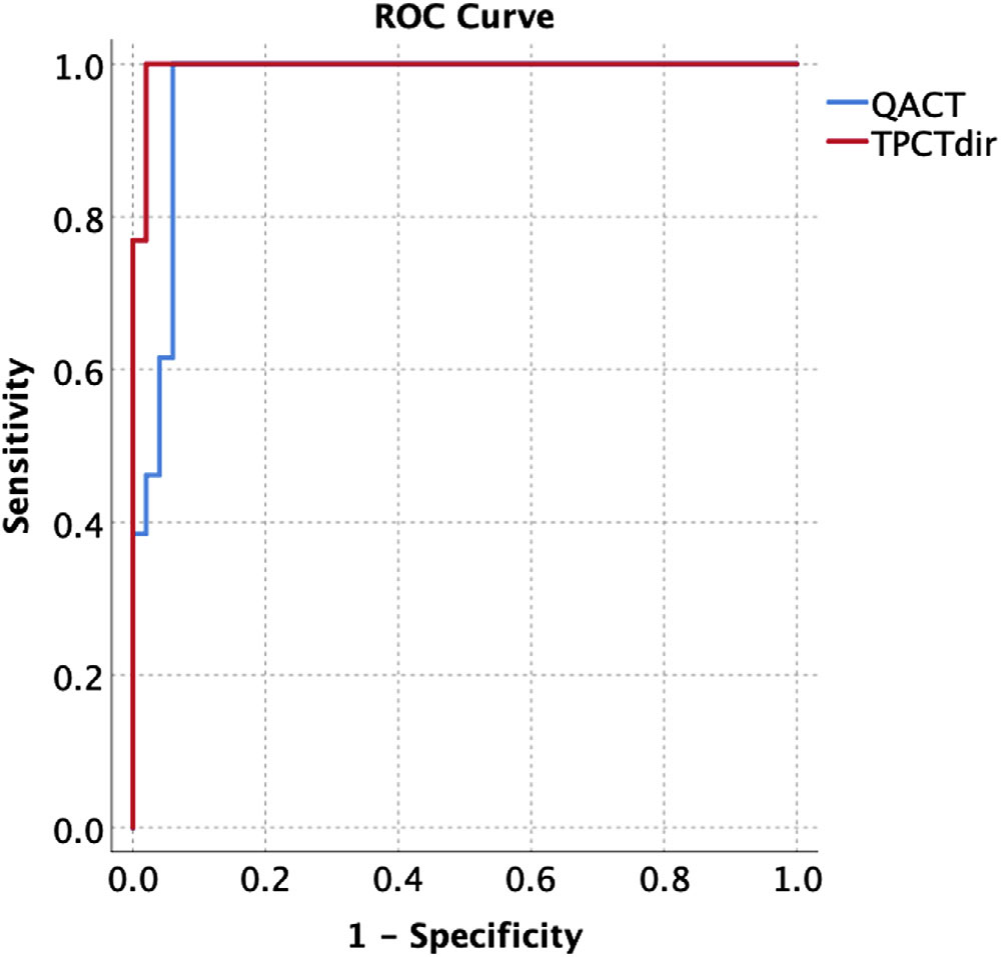
The receiver operating characteristic (ROC) for the quality assurance CT (QACT) and deformed TPCT to CBCT (TPCTdir) in the prediction of replan. The TPCTdir (red line) has a lower false positive rate than the QACT evaluation. Current clinical criteria for replan are based on the clinical target volume (CTV) ΔD95 > 3% in the QACT evaluation

The ROC curves for replan prediction using both TPCTdir and QACT are shown in Figure 6, using the prediction from QACTdir (ΔD95 > 3%) as the reference. The AUCs are 0.969 and 0.995 for the evaluation with QACT and TPCTdir, respectively. For the QACT curve, the first point with a 100% sensitivity occurs at a 2.8% drop in D95. The associated specificity is 94% (false positive rate of 6%). To achieve the same sensitivity with the TPCTdir, a D95 drop of 2.6% must be used, and the corresponding specificity is 98% (false positive rate of 2%).

## 4 DISCUSSION

Proton therapy has superior normal tissue sparing for most H&N treatments, and more patients are being treated in proton facilities (H&N cancer accounts for about 40% of the population at our proton center). However, compared to photon therapy, the highly modulated IMPT plans are significantly more sensitive to patient anatomy changes, such as tumor and normal‐tissue shrinkage due to treatment and weight loss.^1^, ^24^ As a result, more than 30% of H&N cases need to be replanned at least once during their treatments. The current clinical practice for many proton centers is to monitor anatomy changes with weekly or bi‐weekly QACT images, which (1) introduces addition imaging doses and appointment time to the patients, (2) involves significant extra resources like CT simulator and therapist staff time for the QACT procedure, and (3) has low specificity (as shown in Figure 4b and Figure 6). Daily or weekly CBCT scan has been prescribed for most H&N patients in many modern proton and photon treatment centers. Many studies have shown the feasibility of using TPCTdir for dose calculation and plan evaluation with previous photon patients’ image data.^11^, ^13^, ^14^, ^19^, ^25^ For studies with clinical proton patient's data, the implementations were mostly based on open source platforms.^6^, ^10^, ^15^, ^18^ The presented study used commercial software, and all results were based on actual patient data from proton H&N treatments. It could be adapted and implemented for most proton therapy centers.

The statistics for anatomy changes provide a correlation that may predict which plans are worthwhile to investigate. Body volume changes, reflecting weight gain or loss, were significantly higher for the replan group. In more than 87.5% of replan cases, the body volume differences were higher than 100cc, which indicates that this simple parameter could be used as a rough predictor for replan. As shown in Figure 4a, there seems a systematic difference for the body volume changes between QACT and TPCTdir evaluation. We found it was mainly due to the shoulder position differences within the CBCT FOV. At the initial CT simulation, patients’ shoulders were stretched more inferiorly to avoid a beam going through them. At the treatments, the therapists tried their best to reproduce those shoulder positions. However, at the QACT, the shoulder positions were less accurate, and they tend to be more superior (even with the indexed hand holds), with more volume in the CBCT FOV. As a result, there was consistent difference in body volume between CBCT (TPCTdir) and QACT evaluation. For replan patients, the differences were even bigger as for those patients it was difficult to match their shoulder positions at the treatment, and even worse at the QACT acquisition. The AUC for the ROC curve (shown in Figure 4b) from the volume difference of TPCT‐TPCTdir was significantly higher than that of TPCT‐QACT. This again demonstrated that the TPCTdir‐based evaluation, due to its more accurate representation of treatment position, has higher specificity in predicting replan than that based on QACT. The warps associated to the replan group were higher than those of the no‐replan group, but not statistically significant. Both parameters were calculated based on the entire CBCT FOV. The correlation could be stronger if only the change in slices that contain the CTVs are considered, eliminating the anatomy changes far away from the beam path. Another way to improve the prediction power is to include patients’ pretreatment medical characteristics, which is under development by our group.^26^

Overall, the high agreements between the TPCTdir and QACTdir in terms of D95, Dmean, and D1 dose for the CTVs show that a TPCTdir may be used to indicate the necessity of a QACT. In general, when anatomy changes were minimal, and setup for the QACTs was appropriate, the QACT evaluations were more accurate than the TPCTdir evaluation (as seen in the dosimetric comparisons in the last two rows in Table 2), but the difference is small and will not trigger a false negative prediction if a threshold is chosen appropriately for the TPCTdir evaluation. The difference for CTV_High is less than that for the CTV_Low, 0.13% ± 0.69% versus 0.66% ± 1.07% (as shown in Table 2), which represents more uncertainty of TPCTdir at the lower neck and shoulder region for the node CTVs. The same trend holds for the QACT versus the reference QACTdir. For CTV_Low, the variation (standard deviation) is much higher with the QACT (1.56%) than that with the TPCTdir (1.07%), which demonstrates that QACT was associated with higher discrepancy from patient's actual treatment position at the neck and shoulder region. The corresponding evaluation based on QACT may show more false positives, as shown in the ROC curve in Figure 6. The mean dose and D1 evaluated with both TPCTdir and QACT were very close to those from the reference QACTdir. The parotid mean dose was slightly less from TPCTdir, compared to that from the QACTdir. However, the difference is not statistically significant.

The ROC constructed shows the evaluation from TPCTdir is overall more accurate than that from the QACT when evaluation from QACTdir is used as the reference. The QACT is associated with a similar sensitivity while suffering from a lower specificity. For a 100% sensitivity, the threshold of ΔD95 should be 2.8% and 2.6% for the QACT and TPCTdir, respectively. The corresponding false positive rates are 6% and 2%, respectively. Other institutions may use the framework provided in this paper to determine their own thresholds based on their equipment, software, and needs.

As proton therapy is sensitive to setup uncertainty and patients’ anatomy change, therapists spend great effort to align the patient. As kV images are used before the pretreatment CBCT imaging to position the patient, with special attention paid to the shoulder position to warrant accurate doses to the node CTVs, the CBCT image represents much better alignment to the TPCT than that from the QACT, as shown in Figure 1. Moreover, it reflects the actual treatment position. As a result, TPCTdir keeps the fidelity of patients’ treatment position and anatomy change while holding accurate HU for dose calculation. Evaluation based on TPCTdir will reduce the number of CT scans a patient receives, lower their dose, and free time on the CT scanner for other patients.

One limitation of this study is that the accuracy of the dose calculation on the TPCTdir has not been fully validated. Landry et al.^27^ has reported a comprehensive evaluation on CT to CBCT image registration based on a deformable phantom. Without such a phantom, the validation will be limited to the evaluation of DIR from CT to CT images, as one can always deform a TPCT to a QACT and compare the dose calculation from the deformed TPCT to that from the QACT. On the other hand, the DIR accuracy is quite patient‐dependent. DIR software typically provides QA tools, like the Jacobian and deformation vectors visualization, for users to verify the deformation accuracy.

The quality and accuracy of TPCTdir depend not only on the performance of the DIR algorithm and implementation, but also on the skills and experience of the operator. Although Velocity AI (Varian Medical Systems, Palo Alto, California) is used in this study, other commercial software should work well.^28^ We have noticed that the DIR algorithms have difficulties reproducing cavity changes like nasal cavity filling, oral cavity changes (as shown in Figure 3), etc. In these cases, density override can be used to improve the accuracy of generated TPCTdir. In addition, every effort should be made to match the body surface contour from TPCTdir to the CBCT. As shown by other investigators, the WET from TPCTdir may not accurately enough represent doses to a critical serial organ at the distal end of the beam path.^15^, ^18^ In these cases, QACTdir should be used for accurate evaluation.

New development of direct CBCT image correction using deep learning‐based techniques has shown very promising results.^10^ It also shows that the DIR‐based techniques are comparable to the direct image correction methods, with the benefit of being suitable for daily adaptation and less operator‐dependent.

In the future, this work can be expanded to other sites to show the change in target coverage and OAR dose distribution as well. To further develop this technique, a multi‐parameter prediction model is under investigation to improve accuracy and reduce the workload.

## 5 CONCLUSION

The use of actual clinical data from proton H&N treatments showed that the evaluation based on deformed TPCT works well in a clinical setting. The deformation statistics provide a correlation, which may indicate a replan. This work showed that common commercial software packages can adequately perform the DIR of TPCT sets to CBCT for dosimetric evaluation to indicate whether a replan is necessary for patients receiving proton H&N treatment. The ROC analyses from both anatomy volume changes, and dosimetric changes show that TPCTdir‐based evaluations have better specificity than those based on the QACT. Dosimetric thresholds can be derived from such analysis by proton therapy institutions with their own DIR tools and patients’ data.

## CONFLICT OF INTEREST

The authors declare that there is no conflict of interest that could be perceived as prejudicing the impartiality of the research reported.

## AUTHOR CONTRIBUTION

Alex Stanforth prepared the manuscript and collected part of the data. Liyong Lin contributed to the analysis of anatomic changes for the HN patients. Jonathan J. Beitler contributed substantially to the study design and writing of the manuscript. James R. Janopaul‐Naylor participated in this study. He went through many QACT and CBCT images to check the accuracy of the deformed contours. Chih‐Wei Chang conducted statistical analysis. Robert H. Press went through some of the clinical data for this study. Sagar A. Patel contributed to the preparation of the manuscript. Jennifer Zhao helped with the statistical analysis. Bree Eaton and Tian Liu helped with the design of the study. Eduard E. Schreibmann helped with the scripting of some code for the data collection. James Jung and Duncan Bohannon helped collect the data. Xiaofeng Yang worked on the data analysis. Mark W. McDonald provided substantial input on the study design. Jun Zhou initiated this study. He guided and coordinated all the data collection and analysis. He also reviewed and revised the manuscript to the current version.
